# Risco Cardiovascular e Elegibilidade Para Estatina na Prevenção Primária: Comparação Entre a Diretriz Brasileira e a Diretriz da AHA/ACC

**DOI:** 10.36660/abc.20190519

**Published:** 2020-09-18

**Authors:** Fernando H. Y. Cesena, Viviane A. Valente, Raul D. Santos, Márcio S. Bittencourt

**Affiliations:** 1 Hospital Israelita Albert Einstein Hospital Israelita Albert Einstein São Paulo SP Brasil Hospital Israelita Albert Einstein, São Paulo, SP – Brasil; 2 Instituto do Coração Faculdade de Medicina Universidade de São Paulo São Paulo SP Brasil Instituto do Coração (InCor) - Faculdade de Medicina da Universidade de São Paulo, São Paulo, SP – Brasil; 3 Hospital Universitário Universidade de São Paulo São Paulo SP Brasil Hospital Universitário da Universidade de São Paulo, São Paulo, SP – Brasil

**Keywords:** Doenças Cardiovasculares, Dislipidemias, Aterosclerose, Fatores de Risco, Prevenção e Controle, Prevenção Primária, Inibidores de Hidroximetilglutaril/uso terapêutico

## Abstract

**Fundamento:**

Diferenças entre as versões atualizadas da Diretriz Brasileira de Dislipidemias e da Diretriz de Colesterol da *American Heart Association (AHA)/American College of Cardiology (ACC)* quanto à estratificação de risco cardiovascular e à elegibilidade para a terapia com estatina não são conhecidas.

**Objetivos:**

Comparar a categorização de risco cardiovascular e a elegibilidade à terapia com estatina estabelecidas segundo a diretriz brasileira ou a diretriz da AHA/ACC em pacientes em prevenção primária.

**Métodos:**

Nós avaliamos retrospectivamente indivíduos com idade entre 40 e 74 anos sem condições de alto risco, com LDL-c 70 -< 190 mg/dL, sem tratamento com agentes hipolipemiantes, e que passaram por avaliação clínica de rotina. O risco cardiovascular foi estratificado de acordo com a diretriz brasileira e a da AHA/ACC. Os indivíduos foram considerados elegíveis para estatina se os níveis de LDL-c estivessem no mínimo 30 mg/dL acima da meta para o risco cardiovascular (diretriz brasileira) ou se o risco em 10 anos para doença cardiovascular aterosclerótica fosse ≥ 7,5% (diretriz da AHA/ACC). Um valor de p < 0,05 foi considerado estatisticamente significativo.

**Resultados:**

A amostra do estudo consistiu 18525 indivíduos (69% homens, idade 48 ± 6 anos). Entre os indivíduos considerados de risco intermediário ou alto segundo a diretriz brasileira, mais de 80% seriam classificados em uma categoria de risco mais baixo segundo a diretriz da AHA/ACC. Entre os homens, 45% e 16% seriam considerados elegíveis para a terapia com estatina segundo as diretrizes brasileira e da AHA/ACC, respectivamente (p < 0,001). Entre as mulheres, as respectivas proporções seriam 16% e 1% (p < 0,001). Oitenta e dois porcento das mulheres e 57% dos homens elegíveis para estatina com base no critério da diretriz brasileira não seriam considerados elegíveis para estatina segundo o critério da AHA/ACC.

**Conclusões:**

Em comparação à diretriz da AHA/ACC, a diretriz brasileira classifica uma maior proporção dos pacientes em prevenção primária em categorias de risco mais alto e aumenta substancialmente a elegibilidade para estatina. (Arq Bras Cardiol. 2020; 115(3):440-449)

## Introdução

Embora todas as diretrizes para o manejo do colesterol plasmático recomendem realizar a estratificação de risco cardiovascular para guiar a decisão sobre terapia com estatina na prevenção primária, diferentes decisões de tratamento têm sido feitas dependendo da diretriz utilizada.^1-5^ Em um estudo prévio, observamos que uma proporção consideravelmente maior da população em prevenção primária foi considerada elegível a receber estatina com base nas recomendações da V Diretriz Brasileira de Dislipidemias, em relação à diretriz do *American College of Cardiology (ACC)/American Heart Association (AHA)* para o manejo do colesterol de 2013.^6^ Este achado ocorreu como consequência de uma clara discrepância entre a estratificação do risco cardiovascular segundo a diretriz brasileira^1^ e o risco calculado pelas *pooled cohort equations* (PCE), conforme recomendado pela diretriz do ACC/AHA.^3,7^

A Diretriz Brasileira de Dislipidemias foi atualizada em 2017. Algumas modificações foram feitas no processo de estratificação de risco, e uma meta de colesterol da lipoproteína de baixa densidade (LDL-c) foi introduzida para pacientes de baixo risco.^2^ Em 2018, foi publicada uma nova diretriz de colesterol da AHA/ACC, propondo uma nova categorização do risco cardiovascular.^4^ Diferenças entre as versões atuais dessas diretrizes quanto à estratificação de risco e elegibilidade para o uso de estatina na prevenção primária não são conhecidas e são de importância prática para o médico atendente.

Assim, os objetivos deste estudo foram: (1) comparar a estratificação de risco cardiovascular recomendada pela Atualização da Diretriz Brasileira de Dislipidemias (2017) com a recomendada pela Diretriz de Colesterol da AHA/ACC em indivíduos em prevenção primária, sem manifestações clínicas de alto risco cardiovascular; (2) comparar a proporção de indivíduos elegíveis para estatina, segundo critérios dessas duas diretrizes.

## Métodos

### Delineamento e amostra do estudo

Este estudo observacional foi uma análise retrospectiva de indivíduos atendidos consecutivamente como parte de uma avaliação de rotina no Departamento de Medicina Preventiva do Hospital Israelita Albert Einstein (São Paulo-SP, Brasil). A população do estudo corresponde à mesma amostra incluída em nosso estudo prévio^6^ comparando a Diretriz Brasileira de Dislipidemias de 2013 com a Diretriz de Colesterol do ACC/AHA de 2013 (indivíduos que foram atendidos em nosso serviço de 2009 a 2015), além de outros que passaram por uma avaliação até julho de 2018. Os dados foram coletados prospectivamente e reunidos em um grande banco de dados.

Nossa população de interesse foi indivíduos em prevenção primária, sem condições de alto risco, aos quais as diretrizes recomendam o uso de escores de risco para direcionar o uso ou não de estatina.^2,4^ Portanto, excluímos indivíduos com doença cardiovascular aterosclerótica (DCVA) clínica, DCVA subclínica considerada relevante pelo médico atendente, aneurisma de aorta, diabetes mellitus ou doença renal crônica (taxa de filtração glomerular estimada < 60 mL/min), pacientes com LDL-c ≥ 190 mg/dL, LDL-c < 70 mg/dL (que não são considerados candidatos para receberem estatinas segundo a diretriz da AHA/ACC)^4^ e indivíduos em uso de agentes hipolipemiantes. Também excluímos indivíduos com idade menor a 40 anos ou maior que 74 anos para restringirmos a amostra àqueles cuja idade era apropriada para o cálculo do escore de risco cardiovascular global de Framingham (ERF global) e as PCE.^8,9^

### Risco cardiovascular segundo a Atualização da Diretriz Brasileira de Dislipidemias de 2017

Nós calculamos o ERF global,^9^conforme recomendado pela Atualização da Diretriz Brasileira de Dislipidemias de 2017.^2^ As seguintes variáveis são consideradas na estimativa desse risco: idade, sexo, pressão arterial sistólica, uso de medicamentos anti-hipertensivos, colesterol total, colesterol da lipoproteína de alta densidade (HDL-c), diabetes mellitus e tabagismo. Este escore estima o risco de morte coronária, infarto do miocárdio, angina, acidente vascular cerebral isquêmico ou hemorrágico, acidente isquêmico transitório, doença vascular periférica, ou insuficiência cardíaca em 10 anos.

De acordo com a Atualização da Diretriz Brasileira, o risco cardiovascular foi estratificado da seguinte forma:

- ERF global < 5%: baixo risco;
- ERF global entre 5% e 10% (mulheres) ou entre 5% e 20% (homens): risco intermediário;
- ERF global > 10% (mulheres) ou > 20% (homens): alto risco.^2^

### Risco de DCVA segundo a Diretriz da AHA/ACC de 2018

Nós estimamos o risco de DCVA pelas PCE conforme recomendado pela diretriz da AHA/ACC.^4,8^ Esse escore é derivado de coortes dos Estados Unidos da América e considera os mesmos fatores de risco tradicionais do ERF global, além de etnia. Essas equações predizem o risco de eventos duros de DCVA (morte coronária, infarto do miocárdio não fatal, acidente vascular cerebral fatal ou não fatal) em 10 anos.

De acordo com a Diretriz da AHA/ACC de 2018, o risco cardiovascular foi estratificado da seguinte forma:

- Risco de DCVA < 5%: baixo risco;
- Risco de DCVA entre 5% e < 7,5%: risco limítrofe;
- Risco de DCVA entre 7,5% e < 20%: risco intermediário;
- Risco de DCVA ≥ 20%: alto risco.^4^

### Critérios de elegibilidade para terapia com estatina

Nós categorizamos a população do estudo em três categorias de elegibilidade para tratamento com estatina (não elegível, potencialmente elegível e elegível), com base nas recomendações da Atualização da Diretriz Brasileira de Dislipidemias de 2017 ou da Diretriz da AHA/ACC de 2018.

A diretriz brasileira não estabelece recomendações claras quanto ao momento de se iniciar estatina na prevenção primária, mas estabelece metas de LDL-c com base no ERF global: LDL-c < 130 mg/dL, < 100 mg/dL, e < 70 mg/dL para indivíduos de risco baixo, intermediário e alto, respectivamente.^2^ Desta forma, adotamos os seguintes critérios de elegibilidade para receberem estatina, de maneira arbitrária:

- Não elegível: LDL-c abaixo da meta estabelecida para o risco cardiovascular;
- Potencialmente elegível: LDL-c entre a meta para o risco cardiovascular e < 30 mg/dL acima da meta;
- elegível: LDL-c 30 mg/dL ou mais acima da meta para o risco cardiovascular.

A diretriz da AHA/ACC estabelece que indivíduos de risco intermediário ou alto devem ser considerados para iniciarem terapia com estatina, enquanto aqueles de risco limítrofe podem ser considerados em algumas circunstâncias.^4^ Assim, nós consideramos os seguintes critérios para uso de estatina:

- não elegível: risco de DCVA em 10 anos < 5,0%;
- potencialmente elegível: risco de DCVA em 10 anos entre 5,0% e < 7,5%;
- elegível: risco de DCVA em 10 anos ≥ 7,5%.

### Análise estatística

Os dados e as análises foram estratificados por sexo, uma vez que a população do estudo era composta por uma proporção maior de homens que de mulheres. A elegibilidade ao tratamento com estatina também foi analisada por subgrupos pré-definidos, de acordo com o grupo etário e categoria de risco cardiovascular.

As variáveis categóricas foram expressas em números de observações e proporções. As variáveis contínuas foram expressas em médias e desvios padrões ou em medianas e intervalos interquartis se apresentassem distribuição normal ou não-normal, respectivamente. A normalidade foi avaliada visualmente e pelo cálculo da assimetria da distribuição (valores entre -1 e 1 foram considerados consistentes com uma distribuição normal).

Foi usado o teste qui-quadrado ou o teste exato de Fisher para análise, conforme apropriado. Um valor de p < 0,05 foi considerado estatisticamente significativo. Calculamos o coeficiente de correlação de Spearman para avaliar a relação entre o risco determinado pelo ERF global e o risco para DCVA estimado pelas PCE. Usamos o software R e o Microsoft Office Excel para gerenciamento dos dados e construção dos gráficos.

### Aspectos éticos

Este estudo foi aprovado pelo Comitê de Ética em Pesquisa do Hospital Israelita Albert Einstein (CAAE 80925817.5.0000.0071). O Comitê de Ética aprovou uma dispensa do termo de consentimento por escrito devido à natureza retrospectiva das análises.

## Resultados

### População e características basais

**Figura 1:**
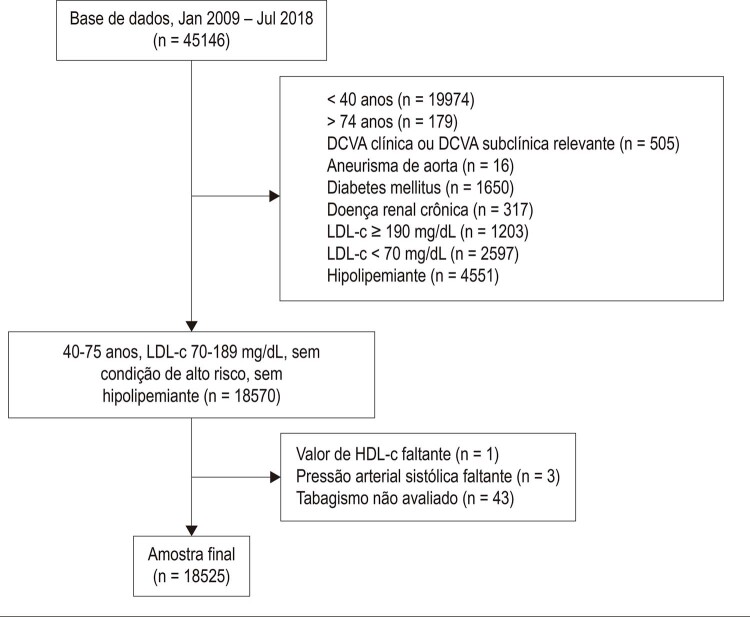
– Fluxograma dos indivíduos incluídos e excluídos do estudo. DCVA: doença cardiovascular aterosclerótica; HDL-c: colesterol da lipoproteína de alta densidade; LDL-c: colesterol da lipoproteína de baixa densidade.

De 45.146 indivíduos identificados inicialmente no banco de dados, 26.621 (59%) foram excluídos, principalmente pela idade inferior a 40 anos (Figura 1). A amostra final consistiu 18.525 indivíduos. Na população do estudo, houve predominância de indivíduos de meia idade, do sexo masculino (69%). A Tabela 1 apresenta as características basais da amostra.

**Tabela 1:** – Características basais da amostra

		Total (n = 18525)	Mulheres (n = 5651)	Homens (n = 12874)
Idade (anos)		48 ± 6	48 ± 6	48 ± 7
IMC (kg/m^2^)		26,9 ± 4,2	25,4 ± 4,4	27,5 ± 3,9
Colesterol total (mg/dL)		202 ± 31	198 ± 30	203 ± 31
LDL-c (mg/dL)		126 ± 27	119 ± 27	129 ± 27
HDL-c (mg/dL)		49 ± 14	58 ± 14	46 ± 11
Triglicérides (mg/dL)		113 (81-162)	89 (67-123)	125 (91-178)
Glicemia de jejum (mg/dL)		87 ± 9	84 ± 8	89 ± 9
Hipertensão arterial		4527 (24)	893 (16)	3634 (28)
Pressão arterial sistólica (mmHg)		119 ± 13	113 ± 13	121 ± 12
Pressão arterial diastólica (mmHg)		78 ± 9	74 ± 8	80 ± 8
Tabagismo		1672 (9)	441 (8)	1231(10)
Risco cardiovascular de Framingham global em 10 anos (%)		5,9 (3,4-9,8)	2,7 (1,8-4,2)	7,6 (5,1-11,7)
Categoria de risco cardiovascular (Atualização da Diretriz Brasileira de Dislipidemias, 2017)	Baixo	7766 (42)	4638 (82)	3128 (24)
	Intermediário	9596 (52)	828 (15)	8768 (68)
	Alto	1163 (6)	185 (3)	978 (8)
Risco de DCVA em 10 anos (PCE, %)		2,2 (1,0-4,5)	0,8 (0,4-1,5)	3,1 (1,7-5,7)
Categoria de risco de DCVA (Diretriz da AHA/ACC, 2018)	Baixo	14498 (78)	5438 (96)	9060 (70)
	Limítrofe	1825 (10)	129 (2)	1696 (13)
	Intermediário	2044 (11)	82 (1)	1962 (15)
	Alto	158 (1)	2 (< 1)	156 (1)

*Dados expressos em média ± desvio padrão, mediana (quartis) ou n (%). AHA/ACC: American Heart Association/American College of Cardiology; DCVA: doença cardiovascular aterosclerótica; IMC: índice de massa corporal; HDL-c: colesterol da lipoproteína de alta densidade - colesterol; LDL-c: colesterol da lipoproteína de baixa densidade; PCE: pooled cohort equations (equações de coorte agrupadas).*

### Risco cardiovascular

**Figura 2:**
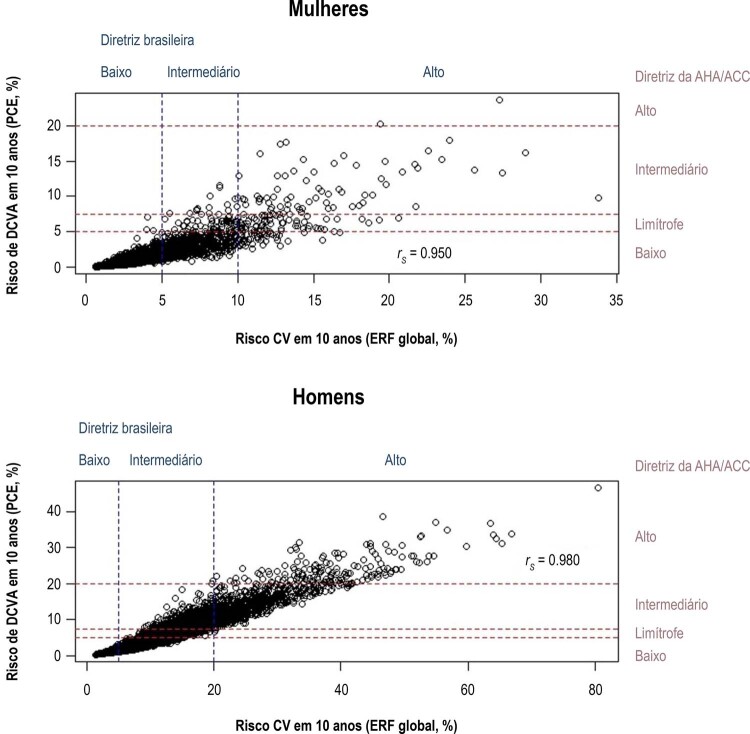
– Correlação entre o risco cardiovascular (CV) global em 10 anos determinado pelo escore de risco de Framingham (ERF) e o risco de doença cardiovascular aterosclerótica (DCVA) em 10 anos estimado pelas equações de coorte agrupadas (PCE, pooled cohort equations), e estratificação de risco segundo a Atualização da Diretriz Brasileira de Dislipidemias de 2017 e a Diretriz de Colesterol da American Heart Association/American College of Cardiology (AHA/ACC) de 2018. rs: coeficiente de correlação de Spearman.

O risco cardiovascular em 10 anos determinado pelo ERF global e o risco de DCVA em 10 anos estimado pelas PCE mostraram-se fortemente correlacionados (Figura 2).

Uma proporção maior da população do estudo seria categorizada como de alto risco de acordo com a diretriz brasileira em comparação à diretriz da AHA/ACC (Tabela 1). Por outro lado, mais indivíduos seriam classificados como de baixo risco pela diretriz da AHA/ACC do que pela diretriz brasileira (Tabela 1). Com exceção de três indivíduos, todos aqueles considerados de baixo risco pela diretriz brasileira seriam também classificados como de baixo risco pela diretriz da AHA/ACC.

Entre os indivíduos de risco intermediário pela diretriz brasileira, uma grande proporção (n = 5.932, 68% dos homens e n = 758, 92% das mulheres) seria estratificada como de risco baixo pela diretriz da AHA/ACC. Somente 1.140 (13%) homens e 15 (2%) mulheres de risco intermediário pela diretriz brasileira seriam classificados nessa categoria pela diretriz da AHA/ACC.

Entre os homens de alto risco segundo a diretriz brasileira, uma minoria (n = 154, 16%) seria classificada como de alto risco pela diretriz da AHA/ACC; a maioria (n = 822, 84%) estaria na categoria risco intermediário. Somente 2 (1%) das mulheres de alto risco segundo a diretriz brasileira estariam na mesma categoria de risco pela diretriz da AHA/ACC, enquanto 45 (24%), 71 (38%) e 67 (36%) mulheres estariam nas categorias baixo risco, risco limítrofe e risco intermediário, respectivamente.

As medianas (quartis) do risco de DCVA em 10 anos pelas PCE nas categorias de risco definidas pela diretriz brasileira foram: 1,2% (0,9-1,5%) entre os homens na categoria baixo risco, 3,7% (2,5-5,7%) na categoria risco intermediário, e 14% (11,6-17,6%) na categoria alto risco; e 0,6% (0,4-1,0%) entre as mulheres na categoria baixo risco, 2,7% (2,0-3,7%) na categoria risco intermediário, e 6,6% (5,1-9,5%) na categoria alto risco.

### Elegibilidade para terapia com estatina

**Figura 3:**
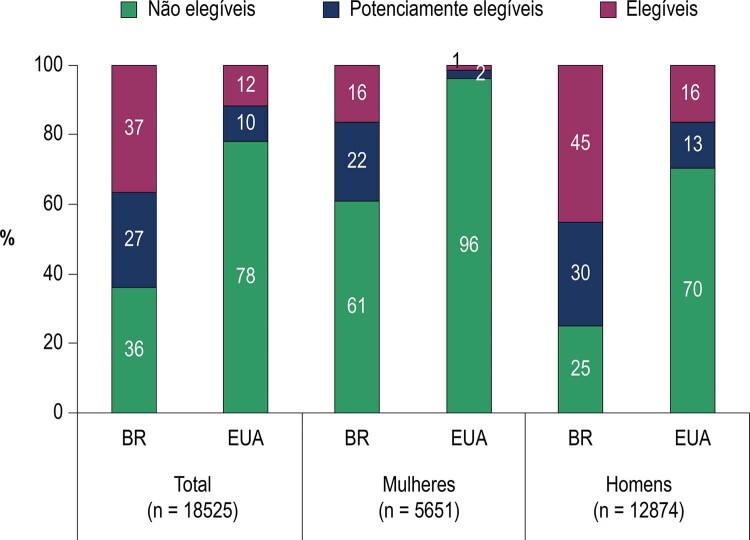
– Proporção de indivíduos não elegíveis, potencialmente elegíveis e elegíveis para terapia com estatina, segundo a Atualização da Diretriz Brasileira de Dislipidemias de 2017 (BR) ou a Diretriz de Colesterol da American Heart Association/American College of Cardiology de 2018 (AHA/ACC) (EUA), na população total e estratificada por sexo. p < 0,001 nos três grupos.

O número de pacientes considerados elegíveis para receberem terapia com estatina seria três vezes maior segundo o critério da diretriz brasileira em comparação ao critério da AHA/ACC (Figura 3). Isso pôde ser observado na maioria dos subgrupos definidos por sexo, idade, e categoria de risco (Figuras 3 a 5 [Fig f04] [Fig f05]). A elegibilidade para terapia com estatina seria maior segundo a diretriz da AHA/ACC somente para homens de alto risco, e de idade mais avançada.

**Figura 4 f04:**
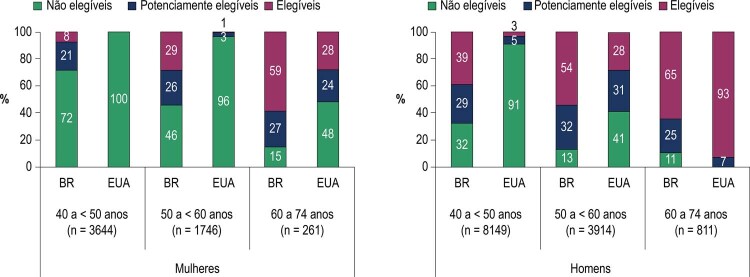
– Proporção de indivíduos não elegíveis, potencialmente elegíveis e elegíveis para terapia com estatina, segundo a Atualização da Diretriz Brasileira de Dislipidemias de 2017 (BR) ou a Diretriz de Colesterol da American Heart Association/American College of Cardiology de 2018 (AHA/ACC) (EUA), por sexo e idade. p < 0,001 em todas as análises dos subgrupos.

**Figura 5 f05:**
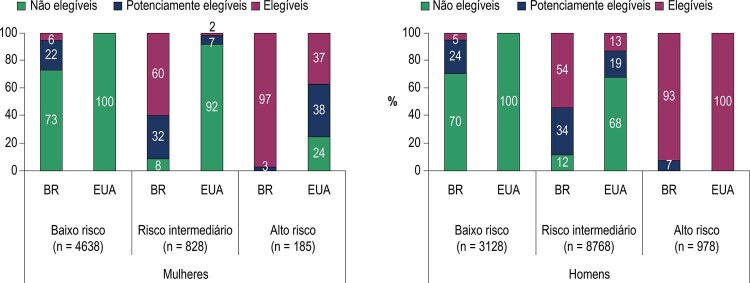
– Proporção de indivíduos não elegíveis, potencialmente elegíveis e elegíveis para terapia com estatina, segundo a Atualização da Diretriz Brasileira de Dislipidemias de 2017 (BR) ou a Diretriz de Colesterol da American Heart Association/American College of Cardiology de 2018 (AHA/ACC) (EUA), por sexo e categoria de risco cardiovascular definido pela Atualização da Diretriz Brasileira de Dislipidemias de 2017. p < 0,001 em todas as análises dos subgrupos.

**Figura 6:**
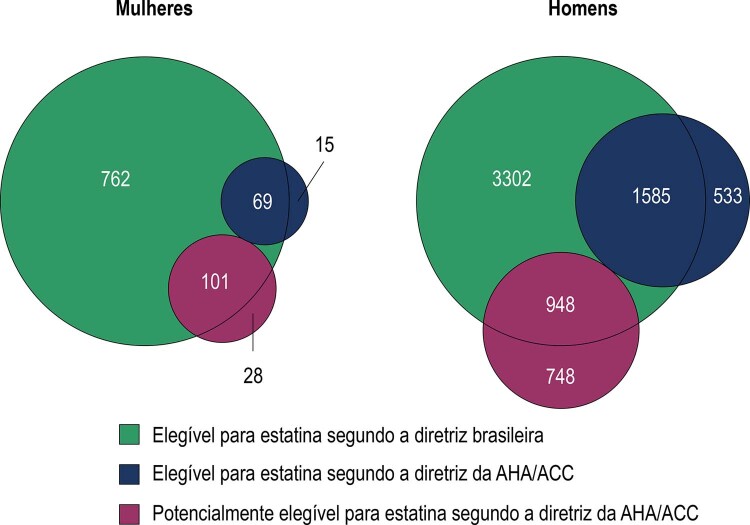
– Diagrama de Venn mostrando as interseções da elegibilidade à terapia com estatina segundo a Atualização da Diretriz Brasileira de Dislipidemias de 2017 (BR) ou a Diretriz de Colesterol da American Heart Association/American College of Cardiology (AHA/ACC) de 2018, por sexo.

Entre as 932 mulheres consideradas elegíveis para terapia com estatina segundo a diretriz brasileira, a maioria (82%) não seria considerada elegível segundo o critério da AHA/ACC; somente 7% seriam consideradas elegíveis e 11% potencialmente elegíveis (Figura 6). Entre os 5835 homens elegíveis para terapia com estatina segundo a diretriz brasileira, 27% também seriam considerados elegíveis pelo critério da AHA/ACC, 16% potencialmente elegíveis e 57% não elegíveis (Figura 6).

## Discussão

Nossos resultados revelam uma nítida discrepância entre a estratificação do risco cardiovascular proposta pela Atualização da Diretriz Brasileira de Dislipidemias de 2017 e a proposta pela Diretriz de Colesterol do AHA/ACC de 2018. Uma grande proporção da população foi classificada como de risco mais elevado pela primeira que pela segunda diretriz. Consequentemente, uma vez que a elegibilidade para terapia com estatina é baseada na estratificação de risco, mais indivíduos seriam considerados elegíveis para terapia com estatina segundo o critério da diretriz brasileira (LDL-c 30 mg/dL ou mais acima da meta recomendada), em comparação com o critério da AHA/ACC (risco em 10 anos para DCVA pelas PCE ≥ 7,5%). Mais da metade dos homens e mais de 80% das mulheres elegíveis para terapia com estatina segundo o critério da diretriz brasileira não alcançariam o limiar de 7,5% de risco para elegibilidade segundo o critério da AHA/ACC.

Diferentes diretrizes recomendam diferentes estratégias para estratificar indivíduos por risco e definir quem devem ser considerados a receberem terapia com estatina (Tabela 2).^2,4,5^ Enquanto a diretriz da AHA/ACC recomenda uma abordagem baseada em risco para orientar o início de estatina na prevenção primária, as diretrizes brasileira e europeia estabelecem metas de concentração plasmática de LDL-c de acordo com o risco cardiovascular. Ainda, enquanto as PCE e a *Systematic Coronary Risk Estimation* (SCORE) recomendadas pelas diretrizes da AHA/ACC e europeia, respectivamente, predizem o risco de desfechos duros, o ERF global recomendado pela diretriz brasileira estima o risco de eventos clínicos duros e moles. Vale ainda ressaltar a “desvalorização” de fatores agravantes (p.ex. síndrome metabólica, níveis elevados de proteína C-reativa ultrassensível, história familiar de doença arterial coronariana prematura) na Atualização da Diretriz Brasileira, bem como uma “valorização” dos fatores agravantes do risco para decisões de tratamento na diretriz da AHA/ACC de 2018 (Tabela 2). De fato, a importância prognóstica bem estabelecida desses fatores corrobora o seu uso na prática clínica.^2,4,5^

**Tabela 2:** – Recomendações gerais das diretrizes de dislipidemia – Brasileira, da *American Heart Association/American College of Cardiology* e europeia

Diretriz	Escore recomendado para estratificação de risco^*^	Recomendações gerais para redução do LDL-c
Atualização da Diretriz Brasileira de Dislipidemias (2017)^2^	Escore de risco global de Framingham^†^	Estabelece metas de LDL-c de acordo com o risco cardiovascular ^‡^
Diretriz de Colesterol da AHA/ACC (2018)^4^	Equações de coortes agrupadas (*pooled cohort equations*)^§^	Estatina recomendada para indivíduos com condições de alto risco, e recomendada ou considerada segundo o risco calculado de DCVA Fatores agravantes de risco (p.ex., LDL-c 160-189 mg/dL, PCRus ≥ 2,0 mg/L, doenças inflamatórias crônicas) e escore de cálcio coronário podem auxiliar na decisão de se iniciar terapia com estatina ou na dosagem do medicamento, especialmente em pacientes de risco intermediário Limiares de LDL-c (em vez de metas) para considerar terapias além de estatinas em subgrupos de risco mais alto //
Diretrizes de Dislipidemia da ESC/EAS (2019)^5^	*Systematic Coronary Risk Estimation* (SCORE)^¶^	Estabelece metas de LDL-c de acordo com o risco cardiovascular^#^

AHA/ACC: American Heart Association/American College of Cardiology; DCVA: doença cardiovascular aterosclerótica; PCRus: proteína C-reativa ultrassensível; ESC/EAS: European Society of Cardiology/European Atherosclerosis Society; LDL-c: colesterol da lipoproteína de baixa densidade. ^*^ O uso dos escores de risco é recomendado na ausência de condições de alto risco (p.ex., DCVA clínica ou LDL-c ≥ 190 mg/dL). ^†^ Estima o risco de morte coronária, infarto do miocárdio, angina, acidente vascular cerebral isquêmico ou hemorrágico, ataque isquêmico transitório, doença vascular periférica, ou insuficiência cardíaca em 10 anos. ^‡^ Metas de LDL-c sob terapia com estatina: < 130 mg/dL (baixo risco), < 100 mg/dL (risco intermediário), < 70 mg/dL (alto risco), < 50 mg/dL (risco muito alto). ^§^ Estimam o risco de morte coronária, infarto do miocárdio não fatal, acidente vascular cerebral fatal ou não fatal em 10 anos. ^//^ Considerar terapia medicamentosa além de estatina se os níveis de LDL-c com estatina em dose máxima tolerada permanecerem ≥70 mg/dL em pacientes com DCVA de risco muito alto ou ≥100 mg/dL em pacientes com hipercolesterolemia primária grave (LDL-c ≥190 mg/dL). ^¶^ Estima o risco de eventos ateroscleróticos fatais. ^#^ Metas de LDL-c: < 116 mg/dL (baixo risco), < 100 mg/dL (risco moderado), redução ≥ 50% do basal e LDL-c < 70 mg/dL (risco alto), redução ≥ 50% do basal e LDL-c < 55 mg/dL (risco muito alto).

O presente estudo atualiza e expande nosso estudo prévio comparando a V Diretriz Brasileira de Dislipidemias e a Diretriz de Colesterol do ACC/AHA de 2013.^6^ Setenta e cinco porcento da amostra da presente análise correspondem aos indivíduos incluídos no estudo anterior; os demais referem-se aos indivíduos atendidos no mesmo serviço mais recentemente. Portanto, nossos resultados permitem uma avaliação do impacto das mudanças na versão atualizada da diretriz brasileira sobre a estratificação de risco e elegibilidade ao tratamento com estatina. Nesse aspecto, observamos uma drástica redução na proporção de indivíduos categorizados como alto risco (mulheres: de 12% para 3% no presente estudo; homens: de 41% para 8%). Esse achado pode ser explicado pela exclusão, na Atualização de 2017, da reclassificação do risco promovida por fatores agravantes.^2^ Mesmo assim, entre os indivíduos considerados de risco intermediário ou alto pela diretriz brasileira, mais de 80% estariam em uma categoria de risco inferior segundo a diretriz da AHA/ACC.

A discordância entre os dois métodos de estratificação já era esperada, uma vez que, embora ambos os documentos recomendem limiares similares para categorização do risco (p.ex. baixo risco se abaixo de 5% em 10 anos), os desfechos considerados nas equações de risco são diferentes , conforme mencionado acima.^2,4^ Assim, um indivíduo com risco de DCVA em 10 anos de 5% segundo as PCE necessariamente tem um risco estimado pelo ERF global em 10 anos maior.

A proporção mais baixa de indivíduos classificados como de alto risco pela Atualização da Diretriz Brasileira de 2017 pode explicar a diminuição na taxa de indivíduos elegíveis ao tratamento com estatina quando comparamos os resultados do presente estudo com os resultados de nosso estudo prévio^6^ (a elegibilidade caiu de 58% para 45% em homens e de 17% para 16% em mulheres). Por outro lado, a elegibilidade à terapia com estatina com base no critério da AHA/ACC, conforme esperado, permaneceu a mesma (17% no estudo prévio e 16% no presente estudo em homens; e 2% no estudo prévio e 1% no presente estudo em mulheres). Portanto, a diferença entre as duas diretrizes (brasileira e da AHA/ACC) reduziu, mas continua bastante alta.

Nossos resultados apontando uma maior elegibilidade para estatina pelo critério brasileiro em comparação à diretriz da AHA/ACC contrastam com um estudo contemporâneo relatando uma elegibilidade à estatina segundo a diretriz do ACC/AHA de 2013 similar àquela estabelecida segundo diretrizes do NICE – *National Institute for Health and Care Excellence* do Reino Unido (2014) e segundo diretrizes da CCS – *Canadian Cardiovascular Society* (2016).^10^ Ainda, mais indivíduos são considerados candidatos à terapia com estatina pela diretriz do ACC/AHA de 2013 do que pelas diretrizes da USPSTF - *U.S Preventive Services Task Force* (2016) ou da *European Society of Cardiology/European Atherosclerosis Society* (ESC/EAS, 2016).^10,11^

Uma maior proporção da população em prevenção primária tratada com estatinas tem o potencial de prevenir mais eventos cardiovasculares,^10^ especialmente em longo prazo. Muitos indivíduos mais jovens elegíveis para estatinas pelo critério brasileiro, mas não pela diretriz da AHA/ACC (uma vez que a idade é o principal determinante do risco para DCVA), apresentam níveis relativamente mais altos de LDL-c e um benefício em longo prazo com estatina comparável ao observado naqueles atualmente recomendados para o tratamento pelo documento da AHA/ACC.^12^

Muitos fatores podem argumentar a favor de um uso mais abrangente de estatinas na população geral: dados genéticos e epidemiológicos que apoiam a hipótese “quanto menor o LDL-c, melhor”;^13^ evidências indiscutíveis dos benefícios das estatinas a partir de ensaios clínicos, mesmo em populações de baixo risco;^14^ perfil de segurança muito bom;^15^ e baixo custo. De fato, mesmo uma estratégia de tratar “qualquer” indivíduo com estatinas tem sido discutida.^16^

Por outro lado, o benefício da terapia com hipolipemiantes depende do risco cardiovascular basal, e a redução do risco absoluto em 10 anos pode ser desprezível em alguns subgrupos da amostra, considerados elegíveis pela diretriz brasileira, mas não pelo critério norte-americano. Um uso mais generalizado de estatina na população geral pode não se justificar com base em uma análise de risco-benefício. A estratégia de se evitar a estatina por alguns anos até que o risco se torne maior pode ser preferida por alguns clínicos e pacientes.

A elegibilidade para terapia com estatina foi maior segundo o critério da diretriz brasileira em todos os subgrupos definidos por idade e sexo, exceto para homens com idade maior que 60 anos, os quais quase sempre alcançaram o limiar de 7,5% de risco para serem considerados elegíveis à terapia com estatina segundo a diretriz da AHA/ACC. Portanto, a recomendação geral de se usar estatina em idosos é uma consequência da diretriz da AHA/ACC ser baseada em risco, e nos parece apropriada, uma vez que os indivíduos de maior risco são aqueles que mais se beneficiam da terapia com estatina. Ainda, a redução de eventos promovida pelas estatinas pode ser observada mesmo quando os níveis basais de LDL-c são relativamente baixos.^17^ O uso de estatina em indivíduos com idade mais avançada também é corroborado por um ensaio clínico randomizado no qual a pravastatina reduziu eventos coronarianos sem interação significativa com o nível basal de LDL-c.^18^

Uma consideração especial deve ser feita às decisões terapêuticas em mulheres. Nós detectamos uma grande disparidade na elegibilidade à estatina entre as duas diretrizes em mulheres de risco intermediário e alto, categorizadas pelo documento brasileiro (Figura 5). Esse achado resulta da enorme discordância na estratificação de risco segundo as diretrizes, a qual está relacionada ao limiar de risco mais baixo (>10% em 10 anos) para categorizar mulheres de alto risco na diretriz brasileira. Essa decisão tomada pelo documento brasileiro seguiu a recomendação da diretriz do AHA de 2011 para prevenção de doença cardiovascular em mulheres,^19^ que não foi adotada por outros países. Uma reavaliação da estratificação de risco em mulheres na diretriz brasileira pode ser sugerida.

Nosso estudo apresenta várias limitações. O critério para elegibilidade à terapia com estatina baseado na diretriz brasileira foi escolhido de maneira arbitrária, uma vez que o documento não especifica quando a terapia deve ser iniciada. No entanto, o pressuposto parece ser uma aproximação razoável da prática clínica de rotina no Brasil, e também apropriado, dada à limitada eficácia das modificações do estilo de vida (p.ex. dieta) em reduzir os níveis de LDL-c no mundo real. Além disso, esse critério foi o mesmo utilizado em nosso estudo prévio,^6^ o que permitiu uma comparação da elegibilidade à terapia com estatina com base na V Diretriz Brasileira ou na Atualização de 2017. Nossos critérios de elegibilidade à estatina foram estabelecidos com base somente no risco calculado; nós reconhecemos que os escores de risco tradicionais não são perfeitos, e outras variáveis não convencionais, tais como os fatores agravantes do risco e a calcificação da artéria coronária, têm um papel importante na tomada de decisão, conforme descrito acima e na Tabela 2.^4,20,21^ Além disso, conforme diretrizes recentes têm enfatizado, a decisão sobre o uso de estatina na prevenção primária deve idealmente ser tomada após uma discussão com o paciente sobre os riscos e benefícios da terapia.^4^ Finalmente, os escores de risco utilizados neste estudo não foram validados ou calibrados na população brasileira. Tal fato é especialmente relevante se consideramos que marcadores socioeconômicos, tal como educação, podem influenciar os desfechos cardiovasculares independentemente dos fatores de risco convencionais.^22^

## Conclusões

Em comparação à diretriz da AHA/ACC de 2018, a Atualização da Diretriz Brasileira de Dislipidemias (2017) classifica uma grande proporção da população em prevenção primária em categorias de risco mais elevado. Consequentemente, a elegibilidade à terapia com estatina é substancialmente maior seguindo-se um critério com base na diretriz brasileira (LDL-c pelo menos 30 mg/dL acima das metas recomendadas) em comparação a uma abordagem baseada na diretriz da AHA/ACC (risco em 10 anos pelas PCE ≥ 7,5%). Pacientes e médicos devem usar o julgamento crítico ao decidirem sobre o início da terapia com estatina na prevenção primária para otimizar a proteção cardiovascular ao longo da vida e, ao mesmo tempo, evitar o excesso de tratamento.
