# Supplementary material for: Risco Cardiovascular e Elegibilidade Para Estatina na Prevenção Primária: Comparação Entre a Diretriz Brasileira e a Diretriz da AHA/ACC
Source: Arq Bras Cardiol. 2020 Sep 18;115(3):440–9. [Article in Portuguese] doi: 10.36660/abc.20190519 (PMC9363097; doi:10.36660/abc.20190519)
Supplement: Translation

# Translation: Cardiovascular Risk and Statin Eligibility in Primary Prevention: A Comparison between the Brazilian and the AHA/ACC Guidelines

**Authors:** Fernando H. Y. Cesena, Viviane A. Valente, Raul D. Santos, Márcio S. Bittencourt

**Affiliations:** 1 Brazil Hospital Israelita Albert Einstein, São Paulo, SP – Brazil; 2 Brazil Instituto do Coração (InCor) - Faculdade de Medicina da Universidade de São Paulo, São Paulo, SP – Brazil; 3 Brazil Hospital Universitário da Universidade de São Paulo, São Paulo, SP – Brazil

**Keywords:** Cardiovascular Diseases, Dyslipidemias, Atherosclerosis, Risk Factors, Prevention and Control, Primary Prevention, Hydroxymethylglutaryl-CoA Reductase Inhibitors/therapeutic use

## Abstract

**Background:**

Differences between the updated versions of the Brazilian Guideline on Dyslipidemias and the American Heart Association (AHA)/American College of Cardiology (ACC) Cholesterol Guideline regarding cardiovascular risk stratification and statin eligibility are unknown.

**Objectives:**

To compare cardiovascular risk categorization and statin eligibility based on the Brazilian guideline with those based on the AHA/ACC guideline in primary prevention patients.

**Methods:**

We retrospectively analyzed individuals aged 40-74 years without high-risk conditions, with LDL-c 70 to < 190 mg/dL, not on lipid-lowering drugs, who underwent routine clinical assessment. Cardiovascular risk was stratified according to the Brazilian and the AHA/ACC guidelines. Subjects were considered eligible for statin therapy if LDL-c was at least 30 mg/dL above the target for the cardiovascular risk (Brazilian guideline) or the 10-year atherosclerotic cardiovascular disease risk was ≥7.5% (AHA/ACC guideline). A p-value < 0.05 was considered statistically significant.

**Results:**

The study sample consisted of 18,525 subjects (69% male, age 48 ± 6 years). Among subjects considered at intermediate or high risk by the Brazilian guideline, over 80% would be in a lower risk category by the AHA/ACC guideline. Among men, 45% and 16% would be statin eligible by the Brazilian and the AHA/ACC guidelines criteria, respectively (p < 0.001). Among women, the respective proportions would be 16% and 1% (p < 0.001). Eighty-two percent of women and 57% of men eligible for statins based on the Brazilian guideline criterion would not be eligible according to the AHA/ACC guideline criterion.

**Conclusions:**

Compared with the AHA/ACC guideline, the Brazilian guideline classifies a larger proportion of primary prevention patients into higher-risk categories and substantially increases statin eligibility. (Arq Bras Cardiol. 2020; 115(3):440-449)

## Introduction

Although all guidelines on the management of blood cholesterol recommend cardiovascular risk stratification to guide the decision about statin initiation in primary prevention, different treatment decisions have been made depending on the guideline used.^1-5^ In a previous study, we observed that a substantially higher proportion of the population in primary prevention was considered statin-eligible based on the recommendations of the V Brazilian Guideline on Dyslipidemias, than on the 2013 American College of Cardiology (ACC)/American Heart Association (AHA) Guideline on the management of blood cholesterol.^6^ There was a clear discrepancy between the cardiovascular risk stratified by the Brazilian guideline^1^ and the risk calculated by the pooled cohort equations (PCE), as recommended by the ACC/AHA guideline.^3,7^

The Brazilian Guideline of Dyslipidemias was updated in 2017. Some changes in the risk stratification process were made, and a target for low-density lipoprotein cholesterol (LDL-c) for low-risk patients was introduced.^2^ In 2018, a new AHA/ACC cholesterol guideline was published, proposing a new categorization of the cardiovascular risk.^4^ Differences between the current versions of the Brazilian and the AHA/ACC guidelines regarding risk stratification and statin eligibility in primary prevention are unknown and are of practical importance for the attending physician.

Therefore, the goals of this study were: (1) to compare the cardiovascular risk stratification as recommended by the 2017 Update of the Brazilian Guideline on Dyslipidemias with that recommended by the 2018 AHA/ACC Cholesterol Guideline in individuals in primary prevention without clinical manifestations of high cardiovascular risk; (2) to compare the proportion of statin-eligible individuals according to the Brazilian guideline and the 2018 AHA/ACC guideline criteria.

## Methods

### Study design and sample

This observational study was a retrospective analysis of individuals consecutively seen as part of a routine evaluation at the Department of Preventive Medicine of the Hospital Israelita Albert Einstein (São Paulo-SP, Brazil). The study population is the same sample included in our previous study^6^ comparing the 2013 Brazilian Guideline on Dyslipidemias and the 2013 ACC/AHA Cholesterol Guideline (individuals who visited our service from 2009 to 2015), in addition to other individuals who underwent a health evaluation up to July 2018. Data were prospectively collected and gathered into a large database.

Our population of interest was individuals in primary prevention, without high-risk conditions, for whom guidelines recommend the use of risk scores to guide statin therapy.^2,4^ Therefore, we excluded subjects with previous clinical atherosclerotic cardiovascular disease (ASCVD), subclinical ASCVD deemed relevant by the attending physician, aortic aneurysm, diabetes mellitus or chronic kidney disease (estimated glomerular filtration rate < 60 mL/min), patients with LDL-c ≥ 190 mg/dL, LDL-c < 70 mg/dL (not candidates for statins according to the AHA/ACC guideline^4^) or taking lipid-lowering drugs. We also excluded subjects younger than 40 years or older than 74 years to restrict the sample to those whose age was appropriate for the calculation of the Framingham general cardiovascular risk score (general FRS) and the PCE.^8,9^

### Cardiovascular risk according to the 2017 Update of the Brazilian Guideline

We calculated the general FRS^9^ as recommended by the 2017 Update of the Brazilian Guideline.^2^ The following variables are considered in this risk estimation: age, gender, systolic blood pressure, use of antihypertensive drugs, total cholesterol, high-density lipoprotein cholesterol (HDL-c), diabetes mellitus, and smoking. This score estimates the risk of coronary death, myocardial infarction, angina, ischemic or hemorrhagic stroke, transient ischemic attack, peripheral vascular disease, or heart failure in 10 years.

According to the Update of the Brazilian Guideline, the cardiovascular risk was stratified as follows:

- general FRS < 5%: low risk;
- general FRS between 5% and 10% (women) or between 5% and 20% (men): intermediate risk;
- general FRS > 10% (women) or > 20% (men): high risk.^2^

### ASCVD risk according to the 2018 AHA/ACC Guideline

We estimated the ASCVD risk by the PCE, as recommended by the 2018 AHA/ACC Guideline.^4,8^ This score is derived from cohorts from the United States of America and considers the same traditional risk factors of the FRS, in addition to ethnicity. The PCE predict the 10-year risk of hard ASCVD events (coronary death, nonfatal myocardial infarction, fatal or nonfatal stroke).

According to the 2018 AHA/ACC Guideline, the ASCVD risk was stratified as follows:

- ASCVD risk < 5%: low risk;
- ASCVD risk between 5% and < 7.5%: borderline risk;
- ASCVD risk between 7.5% and < 20%: intermediate risk;
- ASCVD risk ≥ 20%: high risk.^4^

### Eligibility criteria for statin therapy

We categorized the study population into three categories of statin eligibility (non-eligible, potentially eligible and eligible), based on the recommendations of the 2017 Update of the Brazilian Guideline or the 2018 AHA/ACC Guideline.

The Brazilian guideline does not make clear recommendations on when to initiate statins in primary prevention, but establishes LDL-c targets based on the general FRS, as follows: LDL-c < 130 mg/dL, < 100 mg/dL, and < 70 mg/dL for low-risk, intermediate-risk, and high-risk individuals, respectively.^2^ Accordingly, we arbitrarily considered the following criteria for statin eligibility based on the Brazilian guideline:

- non-eligible: LDL-c below the target for the cardiovascular risk;
- potentially eligible: LDL-c between the target for the cardiovascular risk and < 30 mg/dL above the target;
- eligible: LDL-c 30 mg/dL or more above the target for the cardiovascular risk.

The 2018 AHA/ACC Guideline establishes that individuals at intermediate or high risk should be considered for statin initiation, whereas those at borderline risk may be considered under certain circumstances.^4^ Thus, we considered the following criteria for statin use:

- non-eligible: 10-year ASCVD risk < 5.0%;
- potentially eligible: 10-year ASCVD risk between 5.0% and < 7.5%;
- eligible: 10-year ASCVD risk ≥ 7.5%.

### Statistical analysis

Data and analyses were stratified by sex, since our study population was composed of a higher proportion of men than women. Statin eligibility was also analyzed in pre-defined subgroups, according to age group and cardiovascular risk category.

Categorical variables were expressed as number of observations and proportions. Continuous variables were expressed as mean and standard deviation if normally distributed, or median and interquartile range if non-normally distributed. Normality was assessed by visual inspection of the distribution and calculation of the skewness (values between -1 and 1 were considered consistent with a normal distribution).

The chi-square test and the Fisher exact test, when appropriate, were used in the statistical analyses. A p-value < 0.05 was considered statistically significant. We calculated the Spearman's rank correlation coefficient to evaluate the relationship between the risk determined by the general FRS and the ASCVD risk estimated by the PCE. The R software and Microsoft Office Excel tools were used for data management and graph construction.

### Ethical issues

This study was approved by the Ethics Research Committee of the Hospital Israelita Albert Einstein (CAAE 80925817.5.0000.0071). The Ethics Committee approved a waiver of the written informed consent based on the retrospective nature of the analyses.

## Results

### Population and baseline characteristics

From 45,146 subjects initially identified in the database, 26,621 (59%) were excluded, mainly because of age younger than 40 years (Figure 1). The final sample consisted of 18,525 subjects.

**Figure 1:** – Flowchart of subjects included in and excluded from the study. ASCVD: atherosclerotic cardiovascular disease; HDL-c: high-density lipoprotein cholesterol; LDL-c: low-density lipoprotein cholesterol; SBP: systolic blood pressure.

The study population was characterized by a predominance of middle-aged subjects, mostly (69%) men. Table 1 shows the baseline characteristics of the sample.

**Table 1:** – Baseline characteristics of the sample

		Total (n = 18,525)	Women (n = 5,651)	Men (n = 12,874)
Age (years)		48 ± 6	48 ± 6	48 ± 7
BMI (kg/m^2^)		26.9 ± 4.2	25.4 ± 4.4	27.5 ± 3.9
Total cholesterol (mg/dL)		202 ± 31	198 ± 30	203 ± 31
LDL-c (mg/dL)		126 ± 27	119 ± 27	129 ± 27
HDL-c (mg/dL)		49 ± 14	58 ± 14	46 ± 11
Triglycerides (mg/dL)		113 (81-162)	89 (67-123)	125 (91-178)
Fasting glycemia (mg/dL)		87 ± 9	84 ± 8	89 ± 9
Arterial hypertension		4527 (24)	893 (16)	3634 (28)
Systolic blood pressure (mmHg)		119 ± 13	113 ± 13	121 ± 12
Diastolic blood pressure (mmHg)		78 ± 9	74 ± 8	80 ± 8
Smoking		1672 (9)	441 (8)	1231(10)
10-year Framingham general cardiovascular risk (%)		5.9 (3.4-9.8)	2.7 (1.8-4.2)	7.6 (5.1-11.7)
Cardiovascular risk category (2017 Update of the Brazilian Guideline)	Low	7766 (42)	4638 (82)	3128 (24)
	Intermediate	9596 (52)	828 (15)	8768 (68)
	High	1163 (6)	185 (3)	978 (8)
10-year ASCVD risk (PCE, %)		2.2 (1.0-4.5)	0.8 (0.4-1.5)	3.1 (1.7-5.7)
ASCVD risk category (2018 AHA/ACC Guideline)	Low	14498 (78)	5438 (96)	9060 (70)
	Borderline	1825 (10)	129 (2)	1696 (13)
	Intermediate	2044 (11)	82 (1)	1962 (15)
	High	158 (1)	2 (<1)	156 (1)

*Data expressed as mean ± standard deviation, median (quartiles) or n (%). AHA/ACC: American Heart Association/American College of Cardiology; ASCVD: atherosclerotic cardiovascular disease; BMI: body mass index; HDL-c: high-density lipoprotein cholesterol; LDL-c: low-density lipoprotein cholesterol; PCE: pooled cohort equations.*

### Cardiovascular risk

The 10-year cardiovascular risk determined by the general FRS and the 10-year ASCVD risk estimated by the PCE were shown to be highly correlated (Figure 2).

**Figure 2:** – Correlation between the 10-year general cardiovascular (CV) risk determined by the Framingham risk score (FRS) and the 10-year risk for atherosclerotic cardiovascular disease (ASCVD) estimated by the pooled cohort equations (PCE); and risk stratification according to the 2017 Update of the Brazilian Guideline on Dyslipidemias and the 2018 American Heart Association/American College of Cardiology (AHA/ACC) Cholesterol Guideline. rs: Spearman’s rank correlation coefficient.

A higher proportion of the study population would be categorized as high-risk by the Brazilian guideline, as compared to the AHA/ACC guideline (Table 1). Conversely, more subjects would be stratified as low-risk by the AHA/ACC guideline, in comparison with the Brazilian guideline (Table 1). All but three subjects considered at low risk by the Brazilian guideline would also be classified as low-risk individuals by the AHA/ACC guideline.

Among subjects at intermediate risk by the Brazilian guideline, a large proportion (5,932 [68%] men and 758 [92%] women) would be stratified as low risk by the AHA/ACC guideline. Only 1,140 (13%) men and 15 (2%) women at intermediate risk by the Brazilian guideline would have the same categorization by the AHA/ACC guideline.

Among high-risk men by the Brazilian guideline, a minority (154 [16%]) would also be in the high-risk stratum by the AHA/ACC guideline; most of them (822 [84%]) would be in the intermediate-risk category. Only 2 (1%) of high-risk women by the Brazilian guideline would be in the same risk category by the AHA/ACC guideline, whereas 45 (24%), 71 (38%), and 67 (36%) women would be in the low-, borderline-, and intermediate-risk categories, respectively.

The medians (quartiles) of the 10-year ASCVD risk by the PCE in the risk categories defined by the Brazilian guideline were as follows: 1.2% (0.9-1.5%) of men in the low-risk category, 3.7% (2.5-5.7%) in the intermediate-risk category, and 14.0% (11.6-17.6%) in the high-risk category; and 0.6% (0.4-1.0%) of women in the low-risk category, 2.7% (2.0-3.7%) in the intermediate-risk category, and 6.6% (5.1-9.5%) in the high-risk category.

### Statin eligibility

The number of patients considered eligible for statin therapy would be 3 times higher according to the Brazilian guideline criterion, compared with the AHA/ACC criterion (Figure 3). This could be observed in most subgroups defined by sex, age, and risk category (Figures 3-5 [Fig f04001] [Fig f05001]). Statin eligibility would be higher according to the AHA/ACC guideline only in older and high-risk men.

**Figure 3:** – Proportion of non-eligible, potentially eligible, and eligible subjects for statins, according to the 2017 Update of the Brazilian Guideline on Dyslipidemias (BR) or the 2018 American Heart Association/American College of Cardiology (AHA/ACC) Cholesterol Guideline (USA) in the total population and stratified by sex. p < 0.001 in the three groups.

**Figure 4 f04001:** – Proportion of non-eligible, potentially eligible, and eligible subjects for statins, according to criteria based on the 2017 Update of the Brazilian Guideline on Dyslipidemias (BR) or the 2018 American Heart Association/American College of Cardiology (AHA/ACC) Cholesterol Guideline (USA), by sex and age group. p < 0.001 in all subgroup analyses.

**Figure 5 f05001:** – Proportion of subjects non-eligible, potentially eligible, and eligible for statins, according to criteria based on the 2017 Update of the Brazilian Guideline on Dyslipidemias (BR) or the 2018 American Heart Association/American College of Cardiology (AHA/ACC) Cholesterol Guideline (USA), by sex and category of cardiovascular risk defined by the Update of the Brazilian Guideline on Dyslipidemias. p < 0.001 in all subgroup analyses.

Among 932 women considered eligible for statin therapy according to the Brazilian guideline, the vast majority (82%) would not be eligible according to the AHA/ACC criterion; only 7% would be eligible and 11% potentially eligible (Figure 6). Among 5,835 men eligible for statins by the Brazilian guideline, 27% would also be eligible by the AHA/ACC criterion, 16% would be potentially eligible and 57% non-eligible (Figure 6).

**Figure 6:** – Venn diagram showing the intersections of statin eligibility based on the 2017 Update of the Brazilian Guideline on Dyslipidemias or the 2018 American Heart Association/American College of Cardiology (AHA/ACC) Cholesterol Guideline, by sex.

## Discussion

Our results reveal a clear discrepancy between the cardiovascular risk stratification proposed by the 2017 Update of the Brazilian Guideline on Dyslipidemias and that by the 2018 AHA/ACC Cholesterol Guideline. A large proportion of the population was classified as higher risk by the former than by the latter. As a consequence, once statin eligibility is based on risk stratification, more subjects would be eligible for statin therapy according to a criterion based on the Brazilian guideline (LDL-c at least 30 mg/dL over the recommended target), in comparison with the AHA/ACC criterion (10-year ASCVD risk by the PCE ≥ 7.5%). More than half of men and more than 80% of women eligible for statins based on the Brazilian guideline would not reach the 7.5% risk threshold to be eligible by the AHA/ACC criterion.

Different guidelines recommend different strategies to risk-stratify individuals and decide who should be considered for statin therapy (Table 2).^2,4,5^ While the AHA/ACC guideline recommends a risk-based approach to guide statin initiation in primary prevention, the Brazilian and the European guidelines establish plasma LDL-c targets according to the cardiovascular risk. Moreover, while the PCE and the Systematic Coronary Risk Estimation (SCORE) recommended by the AHA/ACC and the European guidelines, respectively, predict the risk of hard endpoints, the general FRS recommended by the Brazilian guideline estimates the risk of hard and soft clinical events. Also noteworthy is the downgrade of aggravating factors (e.g., metabolic syndrome, elevated high-sensitivity C-reactive protein, family history of premature coronary artery disease) in the Update of the Brazilian Guideline, as well as the upgrade of the so-called risk-enhancing factors for treatment decisions in the 2018 AHA/ACC guideline (Table 2). Indeed, the well-established prognostic relevance of these factors supports their use in clinical practice.^2,4,5^

**Table 2:** – General recommendations of the Brazilian, the AHA/ACC and the European dyslipidemia guidelines

Guideline	Score recommended for risk stratification*	General recommendations for LDL-c reduction
Update of the Brazilian Guideline of Dyslipidemias (2017)^2^	General Framingham risk score^†^	Establishes LDL-c targets according to the cardiovascular risk‡
AHA/ACC Cholesterol Guideline (2018)^4^	Pooled cohort equations^§^	Statin recommended for individuals with high-risk conditions, and recommended or considered according to the calculated ASCVD risk Risk-enhancing factors (e.g., LDL-c 160-189 mg/dL, high-sensitivity CRP ≥ 2.0 mg/L, chronic inflammatory disorders) and coronary artery calcium score may help the decision on statin initiation or statin dosage, especially in intermediate-risk patients LDL-c thresholds (instead of LDL-c targets) to consider therapies beyond statins in higher-risk subgroups//
ESC/EAS Dyslipidemia Guidelines (2019)^5^	Systematic Coronary Risk Estimation (SCORE)^¶^	Establishes LDL-c targets according to the cardiovascular risk#

AHA/ACC: American Heart Association/American College of Cardiology; ASCVD: atherosclerotic cardiovascular disease; CRP: C-reactive protein; ESC/EAS: European Society of Cardiology/European Atherosclerosis Society; LDL-c: low-density lipoprotein cholesterol. ^*^ The use of risk scores is recommended in the absence of high-risk conditions (e.g., clinical ASCVD or LDL-c ≥ 190 mg/dL). ^†^ Estimates the risk of coronary death, myocardial infarction, angina, ischemic or hemorrhagic stroke, transient ischemic attack, peripheral vascular disease, or heart failure in 10 years. ^‡^ LDL-c targets under statin therapy: < 130 mg/dL (low risk), < 100 mg/dL (intermediate risk), < 70 mg/dL (high risk), < 50 mg/dL (very high risk). ^§^ Estimate the risk of coronary death, non-fatal myocardial infarction, fatal or non-fatal stroke in 10 years. ^//^ Non-statin drug therapy to be considered if LDL-c levels in maximally tolerated statin therapy remain ≥70 mg/dL in ASCVD patients at very high-risk or ≥ 100 mg/dL in patients with severe primary hypercholesterolemia (LDL-c ≥ 190 mg/dL). ^¶^ Estimates the risk of fatal atherosclerotic events. ^#^ LDL-c targets: < 116 mg/dL (low risk), < 100 mg/dL (moderate risk), reduction from baseline ≥ 50% LDL-c and < 70 mg/dL (high risk), reduction from baseline ≥ 50% LDL-c and < 55 mg/dL (very high risk).

The present work updates and expands our previous report comparing the V Brazilian Guideline on Dyslipidemias and the 2013 ACC/AHA Cholesterol Guideline.^6^ Seventy-five percent of the sample of the present analysis corresponds to the subjects included in the previous study; the remaining are subjects seen in the same setting more recently. Therefore, our results allow an evaluation of the impact of changes in the updated version of the Brazilian guideline on risk stratification and statin eligibility. In this regard, we observed a drastic reduction in the proportion of individuals categorized as high-risk (women: from 12% to 3% in the present study; men: from 41% to 8%). This finding may be explained by the abolishment, in the 2017 Update, of the reclassification of risk promoted by aggravating factors.^2^ Even so, among subjects considered at intermediate or high risk by the Brazilian guideline, over 80% would be in a lower-risk category by the AHA/ACC guideline.

The disagreement between the two stratification methods was already expected, since although both documents recommend similar thresholds to categorize the risk (e.g., low risk when below 5% in 10 years), the endpoints considered in the risk equations are different, as mentioned above.^2,4^ Therefore, a person with 10-year ASCVD risk by the PCE of 5% has necessarily a higher 10-year risk estimated by the general FRS.

The lower proportion of subjects labeled as high-risk individuals by the 2017 Update of the Brazilian Guideline may explain the decrease in the rate of eligible subjects for statin therapy when we compare the present results with our previous study^6^ (eligibility dropped from 58% to 45% in men and from 17% to 16% in women). Conversely, statin eligibility based on the AHA/ACC criterion, as expected, remained stable (17% in the previous study and 16% in the present study in men; and 2% in the previous study and 1% in the present study in women). Therefore, the difference between the Brazilian and the AHA/ACC guidelines narrowed, but remains very high.

Our findings regarding greater statin eligibility by the Brazilian criterion, compared to the AHA/ACC guideline, contrast with a contemporary study reporting statin eligibility by the 2013 ACC/AHA Guideline similar to that observed using the guidelines from the United Kingdom's National Institute for Health and Care Excellence (NICE, 2014) and the Canadian Cardiovascular Society (CCS, 2016).^10^ Moreover, more individuals are statin candidates by the 2013 ACC/AHA Guideline when compared to the guidance from the U.S. Preventive Services Task Force (USPSTF, 2016) or the European Society of Cardiology/European Atherosclerosis Society (ESC/EAS, 2016).^10,11^

A larger proportion of the population in primary care treated with statins has the potential to prevent more cardiovascular events,^10^ especially in the long term. Many younger individuals eligible for statins by the Brazilian criterion, but not by the AHA/ACC guideline (once age is the main driver of ASCVD risk), have relatively high LDL-c and a long-term benefit from statins comparable to the benefit seen in those currently recommended to the treatment by the AHA/ACC document.^12^

Several factors may argue in favor of a more widespread use of statins in the general population: epidemiological and genetic data supporting “the lower the LDL-c, the better” hypothesis;^13^ unequivocal evidence of benefits of statins from clinical trials, even in low-risk populations;^14^ very good safety profile;^15^ and low cost. Indeed, even a strategy of treating “everyone” with statins has been discussed.^16^

On the other hand, the benefit from lipid-lowering therapy depends on the baseline cardiovascular risk, and the 10-year absolute risk reduction may be negligible in some subsets of our sample which were statin eligible by the Brazilian guideline but not by the North American criterion. A widespread use of statin in the general population may not be justifiable based on a risk-benefit analysis. The strategy of avoiding statin for some years until the risk becomes higher may be preferable by some clinicians and patients.

Statin eligibility was higher by the Brazilian guideline criterion in all subgroups defined by age and sex, except for men over 60 years of age who almost always reached the 7.5% risk threshold for statin eligibility established by the AHA/ACC guideline. Therefore, the general recommendation for statin use in the elderly is a consequence of the risk-based nature of the AHA/ACC guideline and seems appropriate, since individuals at higher risk are those who benefit the most from statin therapy. Also, the event reduction promoted by statins can be seen even when baseline LDL-c levels are relatively low.^17^ Moreover, statin use in older individuals is supported by a randomized clinical trial in which pravastatin reduced coronary events with no significant interaction with the baseline LDL-c level.^18^

Special consideration should be made to treatment decisions in women. We detected a great disparity in statin eligibility between the two guidelines in women at intermediate and high risk, as categorized by the Brazilian document (Figure 5). This finding results from the huge discordance in risk stratification according to the guidelines, which relates to the lower risk threshold (>10% in 10 years) to categorize high-risk women in the Brazilian guideline. This decision made by the Brazilian document followed the 2011 AHA guideline recommendation for prevention of cardiovascular disease in women,^19^ which was not adopted by other guidelines worldwide. A reappraisal of risk stratification among women in the Brazilian guideline may be suggested.

Our study has several limitations. The criterion for statin eligibility based on the Brazilian guidelines was arbitrarily chosen, once the document does not specify when statin therapy should be initiated. However, the assumption seems to be a reasonable approximation of routine clinical practice in Brazil and appropriate due to the known limitations of the efficacy of lifestyle changes (e.g., diet) on lowering blood LDL-c levels in the real world. Moreover, this criterion was the same used in our previous study,^6^ which allowed a fair comparison of statin eligibility based on the V Brazilian Guideline or the 2017 Update. Our criteria for statin eligibility were solely based on the calculated risk; we acknowledge that conventional risk scores are imperfect and other non-traditional variables, such as the risk-enhancing factors and the coronary artery calcification, have an important role in the decision-making process, as noted above and in Table 2.^4,20,21^ Furthermore, as recent guidelines have emphasized, the use of statin in primary prevention should ideally follow a clinician-patient discussion on the benefits and risks of the therapy.^4^Finally, the risk scores used in this study have not been validated or calibrated in the Brazilian population; this is especially relevant if we consider that socioeconomic markers, such as education, may influence cardiovascular endpoints independent of traditional risk factors.^22^

## Conclusions

Compared with the 2018 AHA/ACC Cholesterol Guideline, the 2017 Update of the Brazilian Guideline on Dyslipidemias classifies a great proportion of the population in primary prevention into higher-risk categories. Consequently, statin eligibility is substantially higher according to a criterion based on the Brazilian guideline (LDL-c at least 30 mg/dL over the recommended target), in comparison with an approach based on the AHA/ACC guideline (10-year ASCVD risk by the PCE ≥7.5%). Patients and physicians should use critical judgment when deciding about statin initiation in primary prevention to optimize lifelong cardiovascular protection while avoiding overtreatment.
